# Complete genome sequence of nitrogen-fixing *Rossellomorea marisflavi* strain A1 isolated from the rhizosphere of *Zea mays* L. cultivar Xianyu 335

**DOI:** 10.1128/mra.00930-25

**Published:** 2025-09-29

**Authors:** Hao Li, Qingliu Wu, Xianhong Yong, Xiaoyan Li, Shengyue Tang, Yuxiao Huang, Bin Ni

**Affiliations:** 1 State Key Laboratory of Nutrient Use and Management, College of Resources and Environmental Sciences, China Agricultural University 34752, Beijing, China; Wellesley College, Wellesley, Massachusetts, USA

**Keywords:** *Rossellomorea marisflavi*, diazotrophs, maize, genome analysis

## Abstract

We report the complete genome sequence of *Rossellomorea marisflavi* strain A1, a nitrogen-fixing bacterium isolated from the rhizosphere of *Zea mays* L. (cultivar Xianyu 335) grown in Beijing, China. The genome comprises a single circular chromosome (4,417,820 bp) and one plasmid (500,082 bp).

## ANNOUNCEMENT

Plant-associated microbes play essential roles in nutrient cycling and plant growth promotion. Nitrogen-fixing bacteria (diazotrophs), in particular, are of great interest due to their contribution to sustainable agriculture (1–3). In this study, we isolated strain A1 from the rhizosphere of maize (*Zea mays* L. cultivar “Xianyu 335”), which demonstrated nitrogen-fixing ability and was identified as *Rossellomorea marisflavi*.

**Fig 1:**
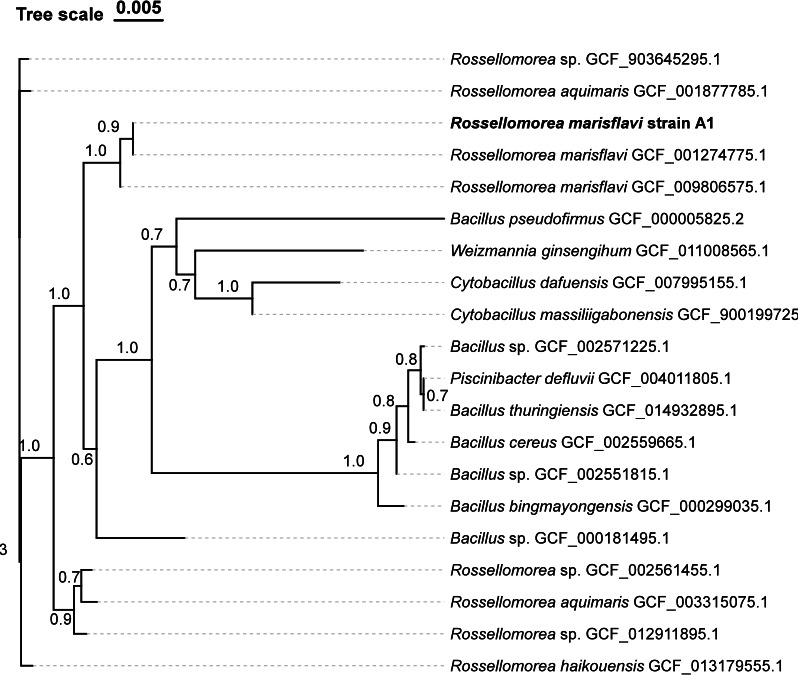
Phylogenetic relationships of *R. marisflavi* strain A1. The tree was constructed using the Neighbor-Joining method in MEGA v6.0 with 20 publicly available reference genomes. Bootstrap values (1,000 replicates) are shown at the branch nodes. Reference genomes were selected by BLAST similarity to strain A1, including the five closest matches and additional genomes chosen at every fifth rank to illustrate diversity; consequently, some genomes from related genera are included. *R. marisflavi* strain A1 is shown in bold.

Rhizosphere samples were obtained by harvesting maize plants grown for 8 weeks in greenhouse conditions using natural soil. Rhizosphere soil was collected by shaking roots in sterile water, and the resulting suspension was serially diluted in 10% Tryptic Soy Broth (TSB) to a 1:2,000 dilution. Aliquots (150 µL) were plated in 96-well plates and incubated at room temperature for 2 weeks (4). Pure culture was isolated by three rounds of continuous streaking. Strain A1 was isolated as a single colony and cultured in 50% TSB for 12 h. Taxonomic identification was performed using 16S rRNA gene sequencing with primers 27F (5′-AGAGTTTGATCCTGGCTCAG-3′) and 1492R (5′-AGAGTTTGATCCTGGCTCAG-3′). BLAST analysis against the National Center for Biotechnology Information (NCBI) NT database (20230605) identified the closest match (identity: 100%) as *R. marisflavi* (GenBank: GCF_001274775.1). The similarity (98.39%) between strain A1 and *R. marisflavi* (GCA_001274775.1) was confirmed by ANI analysis, supporting the species-level classification alongside the 16S rRNA identity. A phylogenetic tree was constructed with the Neighbor-Joining method by MEGA (v6.0) with 20 publicly available reference genomes (Fig. 1).

Genomic DNA was extracted using the Wizard Genomic DNA Purification Kit (Promega, USA). Genome sequencing was conducted using a combination of PacBio RS II Single-Molecule Real-Time (SMRT) and Illumina platforms at Shanghai Majorbio Bio-Pharm Technology Co., Ltd (Shanghai, China). The sequencing methods followed those described in our previous study (5). Illumina sequencing libraries were prepared from the sheared fragments using the NEXTflex Rapid DNA-Seq Kit (Bioo Scientific, USA). For Pacific Biosciences sequencing, the g-tubes (Covaris, USA) method was used to shear the genomic DNA to approximately 8–10 kb fragments. The SMRTbell prep kit 3.0 (Pacific Biosciences, USA) was used to construct the libraries, and a 10 kb insert library was prepared and sequenced on one SMRT cell (Pacific Biosciences, USA).

The genome was determined with PacBio and Illumina (6). Illumina reads were quality-filtered using fastp (v0.20.0) (7), yielding 12,623,180 high-quality reads. Hybrid genome assembly was carried out using Unicycler (v0.4.8), with Pilon (v1.22) used for polishing (8, 9). The complete genome consists of a circular chromosome and one plasmid (500,082 bp, G + C content: 39.40%), both successfully assembled with no rotation. Genome statistics are detailed in Table 1. Gene prediction was performed using Prodigal (v2.6.3) (10) for coding DNA sequences, tRNAscan-SE (v2.0.12) (11) for tRNA genes, Barrnap (https://github.com/tseemann/barrnap) for rRNA detection, and Infernal (v1.1.5) (12) for sRNA. Functional annotation was conducted against the NCBI non-redundant protein database, Swiss-Prot (13), Pfam (14), Clusters of Orthologous Genes (COG) (15), Gene Ontology (GO), and Kyoto Encyclopedia of Genes and Genomes (KEGG) (16).

**TABLE 1:** Overview of genome sequencing, assembly, and annotation statistics for *R. marisflavi* strain A1^*a*^

Parameter	Strain A1
Total no. of raw reads (Illumina)	3412234
Total no. of reads (PacBio)	49487
N50 value (bp)	9891
Avg read length (bp)	9550.57
Genome size (bp)	4417820
G + C content (%)	47.97
Coverage (%)	100
Coding genes in non-redundant protein database (NCBI)	4849
Coding genes in Swiss-Prot database	3556
Coding genes in Pfam database	3861
Coding genes in COG database	3566
Coding genes in GO database	1968
Coding genes in KEGG database	3209

^*a*^ Illumina and PacBio data were combined for hybrid assembly, and genome features were annotated using multiple databases as described in the text. Coverage reported here is based on read alignment, whereas the NCBI-reported coverage is calculated as the total number of bases sequenced divided by the estimated genome size.

The genome contains 5,109 coding sequences, 149 tRNAs, 33 rRNAs, and 99 sRNAs, supporting its metabolic capacity and nitrogen-fixation potential.

## Data Availability

This whole-genome shotgun project has been deposited in GenBank under the accession number CP197480 and CP197481. The associated BioProject and BioSample identifiers are PRJNA1293569 and SAMN50025671, respectively. Raw sequencing reads are available in the NCBI Sequence Read Archive under accession numbers SRR34590790 and SRR34592999. Gene annotation data have been deposited in Zenodo (https://doi.org/10.5281/zenodo.16315965).
